# Environmental Analysis with 2D Transition-Metal Dichalcogenide-Based Field-Effect Transistors

**DOI:** 10.1007/s40820-020-00438-w

**Published:** 2020-04-20

**Authors:** Xiaoyan Chen, Chengbin Liu, Shun Mao

**Affiliations:** 1 Biomedical Multidisciplinary Innovation Research Institute, Shanghai East Hospital, State Key Laboratory of Pollution Control and Resource Reuse, College of Environmental Science and Engineering, Tongji University, 1239 Siping Road, Shanghai, 200092 People’s Republic of China; 2 Shanghai Institute of Pollution Control and Ecological Security, Shanghai, 200092 People’s Republic of China; 3 Department of Materials Science and Engineering, Johns Hopkins University, 3400 N. Charles St., Baltimore, USA

**Keywords:** Environmental analysis, Two-dimensional transition-metal dichalcogenide, Field-effect transistor, Gas sensor, Biosensor

## Abstract

Field-effect transistors (FETs) present highly sensitive, rapid, and in situ detection capability in chemical and biological analysis. Recently, two-dimensional (2D) transition-metal dichalcogenides (TMDCs) attract significant attention as FET channel due to their unique structures and outstanding properties. With the booming of studies on TMDC FETs, we aim to give a timely review on TMDC-based FET sensors for environmental analysis in different media. First, theoretical basics on TMDC and FET sensor are introduced. Then, recent advances of TMDC FET sensor for pollutant detection in gaseous and aqueous media are, respectively, discussed. At last, future perspectives and challenges in practical application and commercialization are given for TMDC FET sensors. This article provides an overview on TMDC sensors for a wide variety of analytes with an emphasize on the increasing demand of advanced sensing technologies in environmental analysis.

## Article Highlights

- Recent advances of two-dimensional (2D) transition-metal dichalcogenide (TMDC)-based field-effect transistor (FET) sensors for environmental analysis are summarized.
- Representative TMDC FET sensors in gaseous and aqueous media analysis are introduced.
- Challenges and future research directions of 2D TMDC FET sensors are discussed.

## Graphical abstract

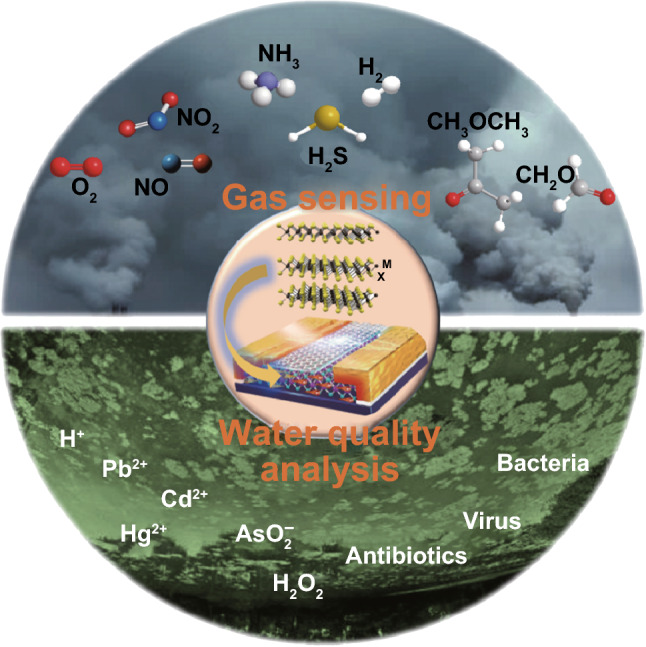

## Introduction

Increasing concerns of living environment and public health lead to a booming of research on new sensing technologies. Sensors based on various mechanisms, e.g., optical sensors based on fluorescence, chemiluminescence, surface plasmon resonance, and electrochemical sensors based on electrochemistry, have been developed to meet the growing demand of environmental analysis [1, 2]. These strategies generally require pre- or post-processing, and chemical reactions in sensing, showing limitations either in rapid and real-time detection or in on-site detection. Field-effect transistor (FET) is an advanced sensing platform relying on electrical signal, which offers rapid sensing capability for a wide range of analytes including gases, ions, organics, and biomolecules [3, 4]. This low-power consumption device works based on the control of transfer performance with a slight gate potential. The electrical properties of FET determine the process of signal conversion in the sensor. Channel material, as the key component in FET sensor, has been studied upon a wide variety of materials, from inorganic semiconductors to organics, from bulk materials to one/two-dimensional (1D/2D) nanomaterials [3–5]. 2D nanomaterials are considered promising channel for FET sensors, and good alternatives of conventional state-of-the-art silicon/metal–oxide–semiconductor field-effect transistors (MOSFETs). They exhibit high potential in miniaturized and low-power transistor due to 2D structure, capable of overcoming limitations from Moore’s law which have long hindered further performance improvement of MOSFETs. 2D nanomaterials are also potentially more sensitive in sensing applications due to their 2D nanostructures that have high specific surface area for dense modification of binding sites [6].

Atomically thin films of transition-metal dichalcogenides (TMDCs), with chemical formula MX_2_ (M = Mo, W, etc., and X = S, Se or Te), are analogues of graphene with layered structure. Over the past few years, TMDCs have drawn wide attention due to their unique properties. Some of the properties make TMDCs even superior to other remarkable 2D nanomaterials including graphene whose zero bandgap and low on/off ratio limit its sensing application and black phosphorus (BP) which has poor chemical stability and durability in ambient condition [7–10]. As the representative TMDC, MoS_2_ shows a high carrier mobility (60 cm^2^/V s at 250 K), a layer-dependent bandgap (1.2–1.8 eV), a high transistor on/off ratio (~ 10^8^), and reasonable environmental stability [11]. Recent reports have demonstrated that 2D MoS_2_ is a desirable channel material in FET sensor with breakthroughs in sensing performance for various analytes including NO_2_ [12–15], NH_3_ [16, 17], chemical vapor [18, 19], metal ion [20–22], small molecule [23–25], as well as biomaterials such as nucleic acid [26–29], protein [30–32], and microorganism [33]. Compared with MOSFET sensors, 2D-TMDC-based FET sensors normally show higher sensitivities due to the 2D nanosheet structure of TMDC. Take gas sensor as an example, MOSFET H_2_ sensors show detection limits in the range of hundreds to thousands ppm [34], and MOSFET NO_2_ sensors show detection limits over ppm level [35]. In contrast, 2D TMDC FET H_2_ sensors have detection limits of several ppm [36–38] and the detection limits of NO_2_ have reached ppb level [39–43].

The basics of TMDC-based FET sensor involve two aspects, the physics and chemistry of 2D TMDCs and the sensing element in FET platform. On one hand, TMDC channel determines the characteristics of FET and plays a vital role in sensing. On the other hand, the sensor performance also depends on the intrinsic properties of FET involving gate (top- or back gate) [44], source, drain electrodes, ohmic or Schottky contact between electrode and channel [45], the physics of dielectric [46], and the doping level of substrate semiconductor [47]. TMDC-based FET sensors also show different characteristics, which relate to the working environment. Gas sensors operate in a relatively clean and chemically inert environment, and the common sensing mechanism is based on direct physical adsorption of gas molecule on TMDC surface, including charge transfer and dipole–dipole interactions [48]. Water contaminant detection normally operates in aqueous media whose sensing mechanism is more complex due to solution chemistry. The aqueous sensing media lead to many research focuses in FET sensor development, including electrostatic interaction, electric double layer, Debye screening effect, etc.[49, 50]. Therefore, classified overview and in-depth analysis of TMDC FET sensors based on working media is of a great significance for the theoretical studies and practical applications of TMDC-based FET sensors.

Till now, some reviews discussed TMDC-based sensors with a focus on particular species of TMDC and a wide range of sensor types, e.g., chemical, electrical, and optical [51–53], have been covered. However, reviews that focus on FET sensing platform are limited, which is greatly needed to promote the research on this important type of electrical sensor. Moreover, most of the reviews focus on sensor structure and detection performance without an emphasize on the impact of working media [10, 54, 55]. Herein, we aim to review recent advances in 2D TMDC-based FET sensors that work in gaseous and aqueous environment. Therefore, this review will introduce the prospective of 2D TMDCs in FET sensor and emphasize the importance of working media. Redox of gaseous analytes and charging characteristics of aqueous analytes are, respectively, adopted as the classification basis from chemistry and electronics. Comprehensive analysis on physics of 2D TMDC and the sensor working principle will be given from TMDC channel structure and properties, surface functionalization, target binding behavior, and signal generation process. At last, the challenges in environment analysis and potential directions for future development of TMDC FET sensor for different sensing applications discussed. We believe this review article will attract considerable attention in environmental science, sensor engineering, and materials science community, potentially posing a wide interest on 2D nanoelectronics.

## TMDC Field-Effect Transistors

### Physical and Electrical Properties of 2D TMDCs

**Fig. 1:**
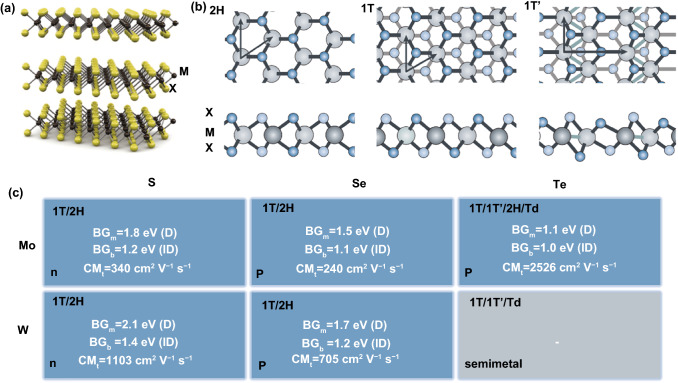
Structure and electronics of 2D TMDCs. **a** Schematic structure of typical MX_2_ materials. Yellow and gray balls indicate chalcogen atoms (X) and transition-metal atoms (M), respectively. Reprinted with permission from Ref. [11], Copyright 2011, Springer Nature. **b** Top and side views of single-layered TMDCs of 2H, 1T, and 1Tʹ phases, showing the primitive unit cell of 2D lattice structure with lattice vectors and the stacking of atomic planes. Reprinted with permission from Ref. [56], Copyright 2017, Springer Nature. **c** Electronics parameters of TMDCs: crystal structure, semiconducting type, bandgap in monolayer (BG_m_) and bulk (BG_b_), and theoretical carrier mobility (CM_t_). *D* direct bandgap, *ID* indirect bandgap. (Color figure online)

TMDC is a large family with chemical formula MX_2_, where M is transition metals from IVB-VIB (Ti, Zr, Hf, V, Nb, Ta, Mo, and W) and X is the chalcogens (S, Se, and Te), up to 24 types overall in theory, though showing various physical and chemical properties [56]. Bulk TMDCs has a layered crystal structure of X–M–X monolayer, it consists of two X atom layers and one M atom layer, in which there are in-plane covalent within each layer and out-of-plane van der Waals interactions for stacking. The lattice structure of a typical TMDC is shown in Fig. 1a, b. The thickness of single-layered TMDCs is ~ 6 to 7 Å and the length of covalent bond between adjacent M is ~ 3.15 to 4.03 Å, which depends on the atomic size of metal and chalcogen [57]. Structurally, 2D TMDC is comparable to graphene and the non-covalent inter-layer interaction is easy to be broken to obtain monolayer structure, which is observed dramatically different from its bulk form material.

Contributed to advances in exfoliation and synthetic techniques, 2D TMDCs (NbSe_2_, MoS_2_, etc.) were initially obtained via mechanical exfoliation strategy like graphene from graphite [58]. In recent years, the preparation methods of 2D TMDC have been developed from mechanical exfoliation to other top-down strategies (e.g., liquid-based ultrasonic exfoliation and lithium ion intercalation) and bottom up methods (e.g., chemical vapor deposition and hydrothermal/solvothermal approach). The specific methodology of TMDCs preparation has been summarized in detail in previous reviews [59, 60].

It is clear that the preparation process makes a big difference on the crystal structure of TMDCs based on the interface chemistry, leading to various lattice types of TMDCs, e.g., trigonal prismatic 2H phase and octahedral 1 T phase [61]. The differences on lattice structure determine their rich electronic band structures, resulting in wide electro-conductibility of TMDCs, from insulator, semiconductors (MoS_2_, MoSe_2_, WS_2_, WSe_2_, etc.), semimetals, conductors to superconductors [56]. From orbital theory, those with *d*-orbitals partially filled show metallic conductivity, while those with *d*-orbitals fully filled are electronically semiconducting [62]. 2H-TMDC (from all VIB- and some IVB-TMDCs) is the thermodynamically stable phase and usually shows semiconducting properties, promising a great fit in FET electronic device. In addition to crystal phase, the band structure of 2H-TMDCs is layer-dependent based on the density functional theory, that is, there is a quantum-drift change at the edge of valence and conduction bands when the layer number increases/decreases [63, 64]. Take MoS_2_ as an example, bulk MoS_2_ has an indirect bandgap of 1.29 eV, while the monolayer 2H-MoS_2_ shows a direct bandgap of 1.9 eV, which is also found in many other TMDCs including WSe_2_, MoSe_2_, WS_2_, ReSe_2_, etc. The physical and electrical properties of typical TMDCs are summarized in Fig. 1c. The direct bandgap of monolayer TMDCs makes it an ideal semiconductor with a tunable layer-dependent band structure, and the Fermi level of TMDCs can be simultaneously adjusted by layer stacking, potentially promising a wide application of 2D TMDCs in electronic device [65].

### Structure of 2D TMDC FET

2D nanomaterials show good potential in miniature and low-power transistor due to their 2D structures and capability of overcoming limitations from Moore’s law [6]. The atomic thickness of 2D material enhances the gate electrostatic control and thus helps suppress the short channel effect. The most studied 2D nanomaterial graphene though has a high carrier mobility of ~ 15,000 cm^2^/V s (at room temperature), its applications in FETs have long been limited by the semi-metallic nature from an absence of bandgap; therefore, in order to open up a bandgap, structure engineering (e.g., nanoribbon and biased bilayer) is applied [66, 67]. Different from graphene, 2D TMDCs have direct bandgap (1.1 to 2.0 eV), which is tunable with adjustment of layer structure. Besides, because of the absence of dangling bonds in 2D crystal, the FET performance degradation on 2D TMDC is well inhibited due to interface states, ensuring a stable device properties [68]. These excellent electrical transport characteristics make 2D TMDC a superior channel material in FET device.

**Fig. 2:**
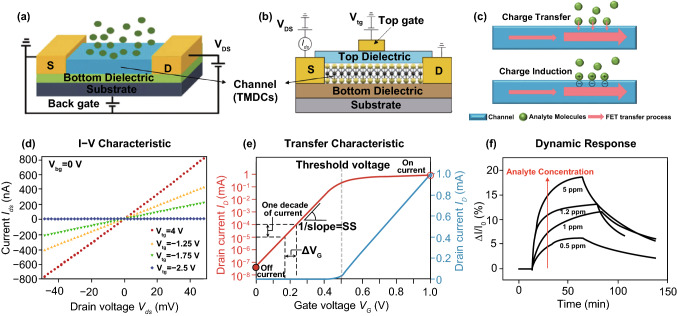
Structure, working principle, electrical characterization, and sensing response of TMDC-based FET sensors. The structures of **a** back gate and **b** top gate FET with TMDC as the channel material. Reprinted with permission from Ref. [3], Copyright 2017, Royal Society of Chemistry. **c** General working principle of FET sensors: charge transfer through the interface of analyte and channel and the charge induction effect between analyte molecule and channel. **d** *I*–*V* characteristic (*I*_ds_ vs. *V*_ds_ curve) of TMDC FET. Reprinted with permission from Ref. [11], Copyright 2011, Springer Nature. **e** Transfer characteristic is obtained by monitoring the drain current with a forward and backward sweep of gate potential. It offers critical parameters of FET device, including on/off ratio, threshold voltage, and subthreshold slope (ss). Reprinted with permission from Ref. [73], Copyright 2011, Springer Nature. **f** Sensing response is obtained by monitoring of the relative drain current change upon the target analyte, varied with the analyte concentration. Reprinted with permission from Ref. [83], Copyright 2012, Wiley

An FET device is composed of source, drain, gate, semiconducting channel, and gate dielectric layer [3]. The typical FET structures include back gate and top gate based on their gate-voltage-dependent FET behavior, as presented in Fig. 2a, b. Both the inherent nature of each component in FET (e.g., electronics of channel material, electrode metal, and dielectric layer) and the geometry between them (e.g., the geometrical width and length of FET) play important roles in electrical properties of the device [69]. With regard to 2D TMDC-based channel materials, the excellent on/off ratio (up to 10^8^) and ideal subthreshold slope (~ 60 mV/decade) were observed in TMDC FETs [70–72]. Besides, studies on FET structure have been carried out to better switch off the device, e.g., applying multiple gates and depositing high-k dielectrics [73, 74].

Electrical properties of FET are the basic for sensing application, including *I–V* characteristic and transfer characteristic. *I–V* characteristic obtained by monitoring source-drain current with a voltage sweep between is important to verify the contact between channel and electrodes (Fig. 2d), yielding the “output” characteristics of the FET. Transfer characteristic is obtained by measuring drain current under a constant drain-source bias (*V*_*DS*_) and a gate potential sweep (Fig. 2e), which provides details of switch behavior including the on/off ratio and subthreshold swings. It can be modeled by equations developed for MOSFETs and organic FETs [69]. In the linear regime, where a low *V*_DS_ is applied, the transfer characteristic can be described by Eq. (1):

1 $${I}_{\text{D}}=\frac{{\varepsilon }_{i}W}{{t}_{i}L}\upmu {V}_{\text{DS}}s\left({V}_{\text{G}}-{V}_{\text{T}}-\frac{{V}_{\text{DS}}}{2}\right),$$

where *ɛ*_*i*_ is the dielectric constant of the insulating gate dielectric layer, *t*_*i*_ is its thickness, *W* and *L* are the width and length of the channel, *µ* is the mobility of semiconducting channel, and *V*_T_ is the threshold voltage. For the saturation regime where a high *V*_DS_ is applied and the channel is at an “off” state, the FET transfer characteristic is described by Eq. (2):

2 $${I}_{\text{D}}=\frac{{\varepsilon }_{i}W}{{2t}_{i}L}\upmu {({V}_{\text{G}}-{V}_{\text{T}})}^{2}.$$

The parameters in these equations show that the FET electrical characterizations are determined by the semiconducting nature of 2D TMDC channel, with influences from electrode morphology and contact interface. Specifically, the inherent nature of 2D TMDC decides the carrier density and mobility, which depend on the type of TMDC and its layer structure. The p- or n-type semiconducting channel can be observed at different surface conditions and the carrier mobility varies with bandgap induced from TMDC as well as the layer number [75]. The dielectric and the structure of electrodes are also important factors for FET performance. For example, an increased charge carrier mobility can be obtained by using high-k dielectric top gate [76], and multiple electrodes and a closer distance between back gate and TMDC surface offer better gate control. In addition, the interface physics, e.g., the work function difference and Schottky barrier between TMDC channel and source/drain electrode as well as their contact condition, determine the contact resistance. The Ohmic or non-Ohmic contact makes a big difference to charge transport and logic applications, which can be analyzed by *I–V* characteristic.

### Working Principle of TMDC FET Sensors

The 2D structure of TMDC offers high specific surface area of channel and the high carrier mobility of 2D TMDC results in ultra-sensitive conductivity via electrostatic perturbation, making 2D TMDC FET an ideal sensing platform. Surface and interface chemistry play an essential role for the transfer characteristics of 2D TMDC. The surface condition includes surface defects, the charged impurity concentration, the local charge distribution, as well as the trapped charges in the substrate [77]. That is, a slight change in surface condition including changes in coulomb scattering and carrier mobility makes big differences on FET device characteristics, leading to a change in device conductivity or resistivity, which can be used as signal for chemical or biological sensing.

In theory, the sensing signal of 2D TMDC FET sensor relies on the change of transfer property of FET induced by analyte molecules [65]. The change of transfer property happens in direct or indirect ways, including physical/chemical adsorption, ions doping, and electrostatic induction via a probe [78]. The working mechanisms of FET sensors normally fall into two categories: charge-modulated mechanism and dielectric-modulated transduction mechanism [79]. Charge-modulated FET sensor works based on surface interactions with analyte molecules, which affect the channel property through charge effect, including charge transfer through the contact interface of analyte and channel and the charge induction effect between channel and analyte molecules, as illustrated in Fig. 2c. Dielectric-modulated FET (DMFET) works based on the change of dielectric constant (or capacitance) of the gate. The dielectric constant change normally happens from an indirect bind of analyte molecules through a detection probe, which leads to a shift in the threshold voltage of FET [80]. DMFETs have been widely studied in biosensors [81, 82], since the recognition of biomolecules usually relies on the detection probe rather than a direct interaction with the channel. Atomic layered structure of TMDC provides rich lattice and edge defects as the direct binding sites for target molecules, and its high specific surface area offers more possibilities for surface functionalization, which allows indirect binding of target molecules through surface functionalized groups.

For quantitative detection, the relative sensing response of FET sensor can be calculated by Δ*I*/*I*_0_, where *I*_0_ is the initial *I*_ds_ before exposing to target analyte and Δ*I* is the change of *I*_ds_ after the analyte being introduced. Figure 2f shows typical dynamic responses of TMDC FET sensor, in which *I*_ds_ is monitored as the signal and Δ*I*/*I*_0_ shows a dependence on the analyte concentration [83]. The charge transfer on FET sensor usually happens upon a direct contact of analyte molecule onto TMDC surface, while the indirect contact (analyte molecule with detection probe) changes the transfer characteristic of FET by the electrostatic charge effect. It should be noticed that some sensor may work based on a combination of charge transfer and electrostatic effect.

Since 2D TMDC materials show high sensitivity to working environment, the sensing medium makes a big impact on the sensing performance. Because of this, the sensing mechanism can be different when the sensor works in air or in water due to the ambient oxygen and water. Therefore, TMDC-based FET sensors working in air, water, or biological environment need to be reviewed separately in order to give a clear understanding on the mechanism. In comparison, due to the complexity of aqueous media and device stability issue, TMDC FET as chemical and biosensor sensors working in aqueous media could be more challenging than those for gas sensing. In this emerging research area, most of the TMDC FET sensors have been fabricated based on MoS_2_ till now, but the use and application of other TMDCs are foreseen and may bring new opportunities of TMDC-based FET sensors for wide sensing applications.

## Sensing Applications

### Gas Sensing

**Fig. 3:**
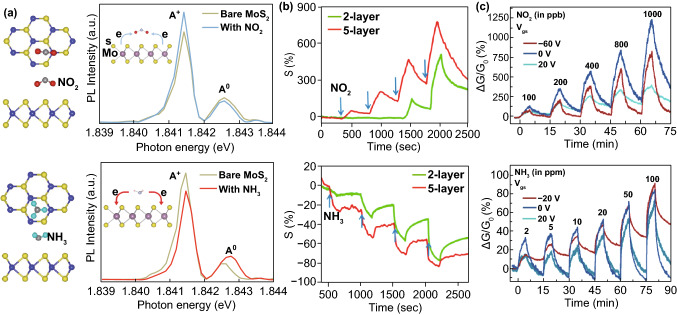
2D TMDC FET sensors for oxidizing and reducing gas detection. **a** Top and side views of favored configurations of NO_2_ and NH_3_ adsorbed on MoS_2_ and in situ PL spectra of MoS_2_ with NO_2_ and NH_3_. The insets present the difference in electron transfer process between gases and channel: NO_2_ and NH_3_ on the surface of MoS_2_ act as electron acceptor and donor, respectively. Reprinted with permission from Ref. [16], Copyright 2015, Springer Nature. **b** Dynamic sensing responses of MoS_2_ FET to NO_2_ and NH_3_ with different layer numbers of MoS_2_. Reprinted with permission from Ref. [89], Copyright 2013, American Chemical Society. **c** Real-time response (relative conductance change) of p-type MoTe_2_ FET sensor to NO_2_ and n-type MoTe_2_ FET sensor to NH3 under different gate biases. Reprinted with permission from Ref. [42], Copyright 2017, IOPscience

Air pollution is a widely concerned global problem, whereas the demand for real-time gas sensing method has promoted research in FET sensors [84, 85]. TMDCs with tunable bandgap, high surface-to-volume ratio, and high adsorption capability for a variety of gases show their advantages as channel material in FET gas sensor [77]. Gas sensing with TMDC FET sensors mainly relies on direct interaction between TMDC surface and gas molecules through physical adsorption, as illustrated in Fig. 3a with NO_2_ and NH_3_ as example [86]. NO_2_ and NH_3_ are the representative of oxidizing and reducing gas, respectively, as the unpaired electrons in NO_2_ and lone electron pair in NH_3_ around N atom determine the tendency to be reduced and oxidized. Changes within TMDC channel in the form of conductance increase or decrease are resulted from charge effect of gas molecule on TMDC, a combination of carriers in channel (i.e., electrons or holes) and redox property of the gas (i.e., oxidizing or reducing). For instance, an exposure of p-type TMDC to oxidizing/reducing gas usually leads to increased/decreased conductivity of the FET channel.

Studies on TMDC FET gas sensor normally focus on the sensitivity, which depends on the strength of gas adsorption. Adsorption energy determines the ability of gas adsorption on TMDC surface, given by *E*_ad_ = *E*_TMDC/gas_ − (*E*_TMDC_ + *E*_gas_) [87], where *E*_TMDC/gas_ is the total energy of the supercell containing both TMDC and a gas molecule, *E*_TMDC_ is the energy of the hosting TMDC supercell, and *E*_gas_ is the energy of the target gas supercell. A negative *E*_ad_ between target gas and TMDC suggests an exothermic adsorption process, which means a voluntary absorption and indicates the feasibility of gas sensing. Reliable analysis of gas sensing behaviors on TMDC channel is generally given by the first-principles calculation based on the density functional theory (DFT), parameters which include the position and orientation of the adsorbed gas molecules on channel [86]. However, TMDC FETs usually show unsatisfied sensitivity and poor selective gas recognition due to the non-specific physisorption. To promote the practical application of TMDC gas sensors, more attention has been paid to the lattice structure optimization and surface modification of 2D TMDC channel for enhanced sensitivity and selectivity. As charge transfer mechanism for gas sensing was verified by in situ photoluminescence spectrum, strategies enhancing electron transfer between gas molecules and TMDC channel are potentially able to improve the sensing performance [16]. Such strategies include crystal structure engineering and material compositing by introducing electroactive material into TMDCs. Since the redox property of gas molecules determines the direction of charge transfer, discussions on TMDC-based FET gas sensors will be given in groups of oxidizing gases, reducing gases, and redox-neutral gases.

#### Oxidizing Gas Detection

Oxidizing gas molecules are oxidizers due to the unpaired electrons and chemically show good affinity to electrons. These gases usually act as electron-acceptors when sensing on TMDC FETs, causing p-doping effects on channel surface by withdrawing electrons from TMDC surface and resulting in source-drain current change. NO_*x*_ (NO_2_ and NO) is one of the typical oxidizing gases that can be detected by TMDC FET sensors. MoS_2_ with single or multi-layered structure shows pronounced sensitivity to oxidizing gases. Studies on mechanically exfoliated MoS_2_ FET gas sensor indicate that the number of MoS_2_ layer and gate bias are two key factors in gas sensing. Monolayer MoS_2_ FET exhibited a rapid response with an unstable electronic output, demonstrated in a report from Zhang et al. for NO sensing. This sensors shows a limit of detection (LOD) of 0.8 ppm with 4-layer MoS_2_ rather than 1-layer MoS_2_ FET [88]. In another work by Late et al. (Fig. 3b), FETs with few-layer MoS_2_ exhibited excellent sensitivity and recovery in NO_2_ sensing [89]. The gate bias study in gas sensing demonstrated that a higher sensitivity can be observed with a more positive gate bias for the sensing of oxidizing gases [42], as shown in Fig. 3c.

NO_2_ works as an electron acceptor and takes electrons from TMDC when adsorbed on the TMDC surface. Since mechanically exfoliated MoS_2_ shows n-type semiconducting, this electron transfer process leads to p-doping on channel surface via electronic effect. Electronic properties of MoS_2_ FETs may vary due to different MoS_2_ preparation processes. The liquid-phase exfoliated [39, 90] and chemical vapor deposition (CVD)-grown MoS_2_ [40] were used as channel in FET sensors for oxidizing gas detection. These methods show advantages in scalable production of large size MoS_2_ nanosheet, but with chemical interactions involved, these methods potentially introduce impurities into MoS_2_, leading to poorer electronic properties than that prepared by mechanical exfoliation, e.g., lower on/off ratio and transfer efficiency [91]. In addition, a thin Al_2_O_3_ passivation layer on the surface of MoS_2_ was demonstrated as a potential strategy for enhanced NO_*x*_ sensing [92]. The sensing mechanism of MoS_2_ FET was investigated by first principle study based on DFT calculation, which depends on adsorption configuration, adsorption energy, charge transfer, and electronics of the channel. It was demonstrated that gas molecules (either charge acceptors or donors) are physisorbed on MoS_2_ with small charge transfer, which is modulated by perpendicular electric field, and oxidizing gases introduce adsorbate states in the bandgap of the host monolayer [86]. Negative adsorption energy of NO_2_ onto MoS_2_ [16], active sites for NO_2_ of $(1\overline{1}0)$ plane of MoS_2_ [93], and slight changes of valence and conduction bands upon introduction of adsorbate state in bandgap by oxidizing gases (O_2_, NO, and NO_2_) [86] were observed. Therefore, exploration on active sites, adsorbate states, and band characteristics change during the gas sensing are needed to further understand the sensing mechanism of MoS_2_ FETs.

In addition to MoS_2_, other layered pristine TMDCs were employed for gas sensing and studies have demonstrated WS_2_ [94], MoSe_2_ [13, 41], WSe_2_ [95–98], MoTe_2_ [42, 43, 99, 100], and NbS_2_ [101] can be used in FET for the detection of O_2_, NO, NO_2_, SO_2_, etc., with a sensing capability of ppm to ppb level. Studies on MoSe_2_ FET sensor suggest that gap state variation is the key mechanism in NO_2_ sensing based on modeling and quantum transport simulation. In NO_2_ detection, the gas adsorption on MoSe_2_ leads to the change in gap states near the valence band and results in an increase in hole current in the off-state regime, contributing to the high sensitivity of the sensor [13]. Research on WSe_2_ FETs has experimentally demonstrated the obvious impacts on electronic structure of TMDC monolayer from the adsorption of oxidizing gases [96]. Based on the sensing mechanism, it explained the higher responses of oxidizing gases against reducing gases [97] and provided strategy for improved recovery performance (e.g., utilizing external thermal energy) [98]. The noise originated from conducting channel [99], the impact of gate bias and Schottky barrier on recovery time [42, 100], and the influence from edge defects of chalcogens [101] were also investigated in MoTe_2_ and NbS_2_ FET platforms.

**Fig. 4:**
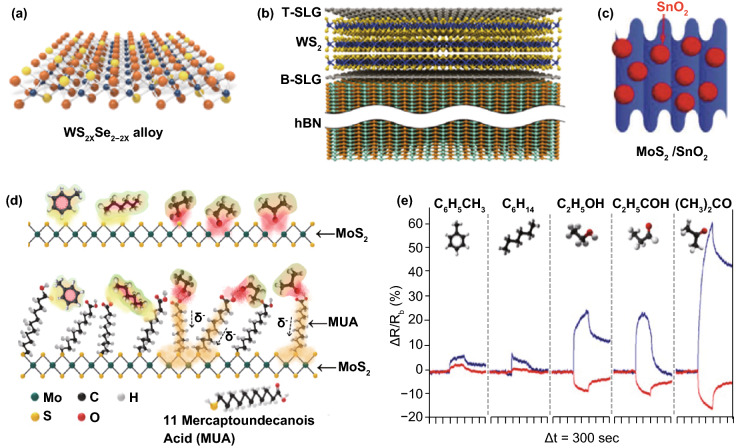
2D TMDC FET sensor for chemical vapors detection. Modifications on TMDC channel to tune the gas sensing performance, including **a** TMDC alloys, **b** heterojunctions, and **c** MoS_2_-nanoparticle composites. Reprinted with permission from Ref. [103], Copyright 2015, American Chemical Society; Ref. [104], Copyright 2017, Royal Society of Chemistry; Ref. [12], Copyright 2015, Wiley. Sensing for various VOCs on pristine and functionalized MoS2 channel: **d** schematic of adsorption-based sensing mechanism and **e** the comparison of real-time sensing responses to different chemical vapors (blue line: pristine MoS_2_, red line: mercaptoundecanoic acid/MUA-modified MoS_2_). Reprinted with permission from Ref. [18], Copyright 2014, American Chemical Society. (Color figure online)

Based on studies of TMDC FET sensor in oxidizing gas detection, functionalization strategies are found to be able to improve sensing performance by means of enhancing gas adsorption, promoting charge transfer efficiency, etc. The modification methods are categorized according to the channel structures, i.e., non-composites or composites. Non-composites refer to alloys (Fig. 4a), while composites are 2D TMDCs that are combined with other 2D materials (e.g., graphene) or lower dimensional materials (e.g., nanoparticles), as illustrated in Fig. 4b, c. TMDC alloys such as WS_2*x*_Se_2−2*x*_ were reported in gas sensing [102, 103], where selenium vacancy defects were observed prominent for the adsorption behavior of oxidizing gas for selective gas sensing. Besides, alloy composition adjusted by controlled sulfurization process showed an optimized sensing performance for NO_2_ (WS_0.96_Se_1.04_), suggesting that alloying is a potential strategy for sensing performance improvement. Construction of hybrid structures within TMDC by employing 2D materials or lower dimensional material (e.g., nanowires and nanoparticle) is an effective strategy to improve the TMDC FET performance in gas sensing. Heterostructures of 2D composites include graphene/WS_2_/graphene [104], MoS_2_/graphene [105], WS_2_/ZnS_2_ [106], and MoS_2_/WS_2_ structures [107]. The heterostructure promotes sensing performance for oxidizing gas and MoS_2_/WS_2_ heterostructure showed a dramatically enhanced sensing performance for NO_2_ compared with pristine MoS_2_ or WS_2_ nanosheet [107]. In addition, the decoration of 1D or 0D materials in TMDC provides more possibilities to improve sensing performance and the detailed mechanisms behind the performance enhancement depend on their unique hybrid structure. For instance, Schottky barrier was suggested as the reason for enhanced NO_2_ detection with Pt nanoparticle-modified MoS_2_ [83]. SnO_2_ and WO_3_ nanocrystals were demonstrated contributing to the selective detection of NO_2_ with MoS_2_ and WS_2_ [12, 38]. Ag nanowire-functionalized WS_2_ exhibited enhanced sensitivity and recovery property for NO_2_ due to the n-doping effect and low surface energy of Ag [15]. Moreover, the observation of excellent sensing performance of metal-organic framework (MOF) ZIF-67-modified WS_2_ potentially opened a new area for functionalized TMDC channel [108].

#### Reducing Gas Detection

Reducing gases are those having lone electron pair in outermost electron layer of the component atoms, working as reductant and showing tendency of losing electrons or accepting protons. Reducing gas performs as electron donor when sensing with FET platform, and once reducing gas is introduced onto the 2D TMDC channel, its electrons are transferred to the conduction band of TMDC, leading to a resistance change of the sensor.

Similarly, MoS_2_ is the most studied TMDC material in FET sensor for reducing gas (e.g., NH_3_) sensing. With a negative *E*_ad_ for NH_3_ (− 0.25 eV) [86], 2D MoS_2_ shows superiority in sensing of reducing gases with voluntary adsorption behavior and generally exhibits a resistance decrease based on the charge transfer mechanism [16]. Impacts from layer number [83, 89] and layer direction [109] were investigated on sensing performance. Multilayer MoS_2_ shows higher sensitivity and better recovery for NH_3_ over the single-layer counterpart, as well as better stability [89]. For the layer direction, vertically aligned MoS_2_ nanosheets exhibit higher sensing performance compared to horizontally aligned nanosheets, owing to the enhanced gas adsorption in edge sites [109]. Besides, compared with NO_2_, CVD-grown monolayer MoS_2_ shows a decreased Schottky barrier and contact resistance in sensing of NH_3_, giving a detection limit of 1 ppm (compared with 20 ppb for NO_2_) [40]. The decreased sensitivity is resulted from the electric field built by gate potential at contact interface that repels the electron from NH_3_ to channel, indicating better sensitivity for NH_3_ without applying gate voltage. Therefore, studies on electric filed and bias effect on MoS_2_ FET are critical to enhance the sensing performance of reducing gases [86, 110].

In addition to MoS_2_, TMDC-based channel materials including WS_2_, MoSe_2_, and MoTe_2_ have been demonstrated in FET sensors for reducing gas sensing. WS_2_ has a direct bandgap of 1.8 to 2.1 eV, high electron mobility up to 234 cm^2^/V s at room temperature, and an ambi-polar field-modulation behavior, indicating a more promising potential than MoS_2_ in FET gas sensing [87]. It has been experimentally demonstrated to have high sensitivity at room temperature with low LOD (1.4 ppm) for NH_3_ sensing based on physical adsorption mechanism [94, 111] and a better recovery performance [112]. WSe_2_ [96–98] and MoTe_2_ [42, 43, 113] showed their unique gas sensitivity (down to 3 ppb) based on the different bond length due to the atomic radii differences of chalcogens. In these studies, gate bias and Schottky junction were also found to be critical factors in sensing performance [42, 43] and poor recovery was overcome by applying external thermal energy for Schottky barrier tuning [98].

Structure engineering and functionalization strategies have also been adopted to enhance the sensing performance of TMDC FET for reducing gases. Layered heterostructures of graphene/WS_2_/graphene formed with van der Waals force was reported for NH_3_ detection, and the feasibility of selective NH_3_ detection in mixed gases with this heterostructure was also demonstrated [104]. In contrast, doping has been commonly used in TMDC for reducing gas sensing, where noble metallic nanoparticles (NMNPs) and nanocrystals (NCs) were used based on different mechanisms. NMNPs including Pt, Pd, Au, and Ag are able to adjust the electrophilicity/nucleophilicity of the TMDC sensing surface and its affinity to target gas molecules [114, 115]. The decoration of nanocrystals offers extra chemical or electronic activity for reducing gas sensing, e.g., the modification of WO_3_ onto WS_2_ [38] and Pd–SnO_2_ onto MoS_2_ [116]. Further studies on TMDC alloys and 2D composites as FET channel are required to better understand the sensing properties of reducing gas, and doped TMDC structures with new adsorption behavior at phase interface deserve deeper investigation for gas sensing applications.

#### VOCs’ and Other Gases’ Detection

Non-redox gases in the air can also be harmful due to their unique biological toxicity or environmental side effects. Detection of these toxic gases, typically volatile organic compounds (VOCs), with TMDC-based FET sensors has been demonstrated in recent years. Studies have demonstrated the superiorities, especially high specificity, of TMDC in sensing of VOCs (including ethanol, acetonitrile, toluene, chloroform, methanol, etc. against other 2D materials [117–119]. Mechanically exfoliated MoS_2_ showed rapid response but different detectability for VOCs including methylbenzene, hexane, ethanol, acetone, and trimethylamine. As shown in Fig. 4d, e, surface functionalization (e.g., 11-mercaptoundecanoic acid/MUA) on MoS_2_ was observed exhibiting adjustment effect between sensitivity and selectivity by changing configuration of gas adsorption [18]. Dynamic studies on VOCs’ sensing showed that Schottky barrier was important for selective detection (labile nitrogen detection) and recovery [120] in addition to the influence from inherent property of chalcogenides in TMDC [118]. Other TMDCs such as WS_2_ (1T-WS_2_ and n-type multilayer WS_2_) [94, 121] and MoTe_2_ [19] also showed their potential in chemical vapor detection for methanol, ethanol, and ketone compounds with high specificity and stability.

Modifications on TMDC-based channel material include forming heterostructure and chemical functionalization. Besides MUA functionalization on MoS_2_ with sensing performance adjustment capability as discussed above [18], a thin layer of hexagonal boron nitride (h-BN) onto MoS_2_ as a heterostructure also showed positive effects on selective sensing for chemical vapors [122]. The heterostructure and chemical functionalization have a big research space for TMDC-based FET sensors in chemical vapor sensing. Since there are a wide range of chemical vapors with different physical and chemical characteristics and that the reported works were normally carried out under single gas environment, it is still far from achieving molecular recognition till now. Therefore, further studies for sensing performance improvement are required for practical use especially in selective detection of chemical vapors.

### Water Quality Analysis

Water contaminants detection conventionally relies on instrumental methods including spectroscopy and chromatography (e.g., gas chromatography and high-performance liquid chromatography-related methods), which have limitations in rapid and in situ detection [123, 124]. FET sensors provided a new methodology to meet the demanding for real-time water quality monitoring and rapid detection, playing an important role in next-generation environment analysis. In addition to the superiorities of TMDCs in FET gas sensors summarized before, the high mechanical strength and flexibility (i.e., high Young’s modulus of 0.33 ± 0.07 TPa) extended their applications in a more complex and stressed environment such as under water [125]. However, FET sensor working in water show a different sensing mechanism and the operational module becomes important due to the aqueous impact on the mobility of TMDC [126], whereas impact from ions in water needs to be considered. Positively and negatively charged ions in water are comparable to oxidizing and reducing gases based on charge donor–acceptor theory. The cations/anions perform as the electron acceptor/donor and cause a p-/n-doping effect with direct charge transfer. At the same time, electrostatic interactions with ion hydration in water lead to a different sensing mechanism, making it possible to induce an inverse doping effect upon an indirect binding. Therefore, water quality sensing can be complex due to the different sensing mechanisms and impacts from water medium. This section will discuss TMDC-based FET sensors in water quality analysis with focuses on ions (cations and anions) and non-ionic molecules.

#### Detection of Ions

Most of the dissolved water contaminants are in the form of ions with positive or negative charges, i.e., cations and anion. Since research in this field is still at the early stage, there are limited studies and applications of TMDC-based transistors for ion sensing. By discussing their generalities and differences in sensing mechanism and device construction, it may give an insight in the research of TMDC FET sensors in water contaminants detection.

**Fig. 5:**
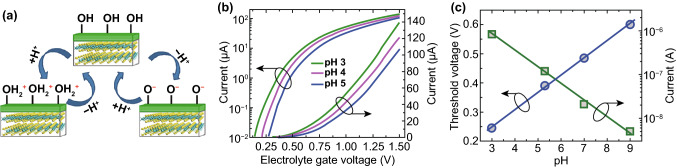
2D TMDC FET pH sensors. **a** Illustration of surface chemistry of channel in pH sensing: the protonation/deprotonation of OH group on the dielectric surface at a low/high pH leads to a positive/negative surface charge on channel. **b** Drain current of the sensor is plotted as a function of gate voltage with different pH values. **c** Changes of threshold voltage (left axis) and current (right axis) of MoS_2_ FET at a wide range of pH (3–9). Reprinted with permission from Ref. [81], Copyright 2014, American Chemical Society

H^+^ and metal ions, especially heavy metals with bio-toxicity, are the main concerned cations in water, the levels of which are important water quality parameters. The capability of TMDC FET sensors for water quality analysis was first demonstrated on MoS_2_ platform for pH sensing [81]. The concentration of H^+^ influences the protonation and deprotonation behavior of OH groups on sensing surface and changes the dielectric surface charges, as illustrated in Fig. 5a. This pH-dependent surface charging is the origin of transfer characteristic changes along MoS_2_-based channel, leading to high current responses at a particular gate bias (Fig. 5b). The testing results showed a linear shift (59 mV/pH) over a wide pH range (3–9), as seen in Fig. 5c [81]. In FET theory, a smaller subthreshold swing indicates a higher sensitivity for pH change due to the gating effect, and the pH sensing performance in MoS_2_ FET is excellent due to its 2D structure and pristine interface. HfO_2_ and Al_2_O_3_/HfO_2_ were then fabricated as the gate dielectrics on the top of MoS_2_, giving a highly linear, stable, and repeatable responses [127]. Besides, a sandwich structure of trilayer made up of MoS_2_/WS_2_/MoS_2_ also showed good subthreshold performance, as well as Nernst sensitivity with high sensing responses for H^+^ (pH of 4–8) [128]. Hence, TMDC FETs are promising sensors for pH sensing in water.

**Fig. 6:**
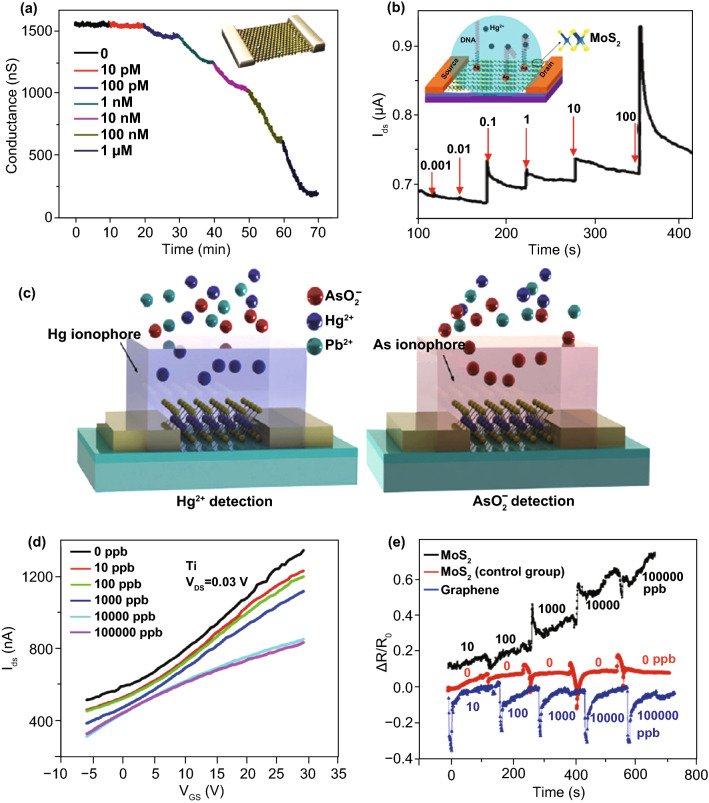
2D TMDC FET ion sensors. **a, b** MoS_2_-based FET Hg^2+^ sensors. Real-time conductance change signals of **a** bare MoS_2_ and **b** DNA-functionalized MoS_2_ at different concentrations of Hg^2+^. Reprinted with permission from Ref. [20], Copyright 2015, Springer Nature; Ref. [21], Copyright 2016, American Chemical Society. **c** Schematic of sensor structure by employing ionophore onto MoS_2_ channel. **d** Transfer curve shifts and **e** real-time sensing responses of MoS_2_ FET at different concentrations of AsO_2_^−^. A higher detectability for AsO_2_^−^ than that of graphene channel is suggested by a direct comparison. Reprinted with permission from Ref. [22], Copyright 2016, AIP Publishing

Metal ion sensing on TMDC-based FET sensors has also been demonstrated with MoS_2_ for Hg^2+^, Pb^2+^, As^3+^, etc. Owing to the affinity between Hg^2+^ and S atoms on MoS_2_ layer surface, the first MoS_2_ FET sensors for Hg^2+^ detection was constructed based on a direct binding of Hg^2+^ onto mechanically exfoliated few-layer MoS_2_ [20]. As the electron acceptor, Hg^2+^ caused p-type doping and reduced electron concentration in MoS_2_. The n-type MoS_2_ FET responded to Hg^2+^ of different concentrations with conductance decreases as shown in Fig. 6a. Besides, Hg^2+^ detection on MoS_2_ FET platform was also achieved with DNA functionalization on the channel surface via gold nanoparticle as the linker [21]. The sensing mechanism of which is quite different from a direct binding of Hg^2+^, but relying on the formation of T–Hg^2+^–T chelates (T is thymine). The sensing response of DNA-functionalized MoS_2_ FET is shown in Fig. 6b. This sensor showed a highly selective Hg^2+^ detection with a fast response (1–2 s) and low detection limit (0.1 nM). Despite Hg^2+^ showing an inherent affinity to MoS_2_, other metal ions’ sensing with TMDC FET sensors relies on sensing probe modified on TMDC surface due to the absence of specific affinity. A recent report has achieved selective detection of Na^+^, Hg^2+^, Cd^2+^, and Pb^2+^ at trace concentration with MoS_2_ FET sensors by using different types of ionophore as the probe [129]. The co-effect of functionalization and surface stabilization with ionophore provides a solution for metal ion sensing on those chemically unstable TMDCs.

Anions in water include nutrient ions and those containing poisonous elements. Anions detection with 2D nanomaterial FET sensors was reported; however, few were constructed on TMDC-based FET platforms. A sensor structure with ionophore was constructed for the sensitive and selective detection of AsO_2_^−^ on MoS_2_ FET; the sensor structure and typical responses are shown in Fig. 6c–e. This sensor showed a very low detection limit down to 0.1 ppb with no influence from large Schottky barrier [22]. In another study, organic functional group carboxylated polypyrrole (CPPy) was modified onto MoS_2_ by vapor deposition [130], and it selectivity grasped AsO_3_^3−^ by conjugation effect, achieving a rapid detection within 1 s and an LOD down to 1 pM. Overall, functionalization of TMDC can either rely on physical deposition based on the superior adsorption capability of 2D materials or a chemical modification via a transition-metal- or chalcogenide-affinitive linker.

In addition to surface functionalization with sensing probe, which determines charge transfer process, device construction is also critical due to influences from gating effect, by means of applying back or top gate. Charged molecules binding on TMDC channel surface are able to induce an effective gating field, which plays an important role in balancing the charge transfer and gating effect, and thus influence the sensing signal. Research on TMDC-based FET sensors for ion detection including channel material, sensing surface chemistry, as well as device structure and electronics is of great value for sensor development and their broader applications.

#### Detection of Non-ionic Molecules

In addition to anions and cations with positive and negative charges, those electrically neutral molecules dissolved in water may also pose threats on aquatic environment as well as human health, such as industrial wastes or pharmaceutical and personal care products (PPCPs). The sensing mechanism of these non-ionic water contaminants on TMDC FETs can be different from those charged molecules and thus requires novel sensor structure design and methodology. Specifically, the lack of distinct tendency of direct charge donation or acceptance in non-ionic molecule sensing makes TMDC FET sensors highly depend on surface functionalization. Though pristine TMDC FETs have shown sensitive response to some small neutral molecules such as hydrogen peroxide [24], studies on macromolecule detection are more significant for the detection of wider range of chemicals [131].

PPCPs including antibiotics and some other pharmaceuticals as emerging environmental threats have been successfully detected on TMDC-based FET sensors [132, 133]. From sensing capacity and selectivity, biomaterials were proposed and demonstrated in these studies as effective detecting probe, which were functionalized on TMDC surface. For instance, a water-soluble variant of μ-opioid receptor was functionalized onto MoS_2_ via an atomic length nickel-mediated linker, achieving a sensitive detection (3 nM level) of enkephalin in water [30], while Cu^2+^-DNA-modified MoS_2_ exhibited excellent detection capability for doxorubicin-like molecules [132].

**Fig. 7:**
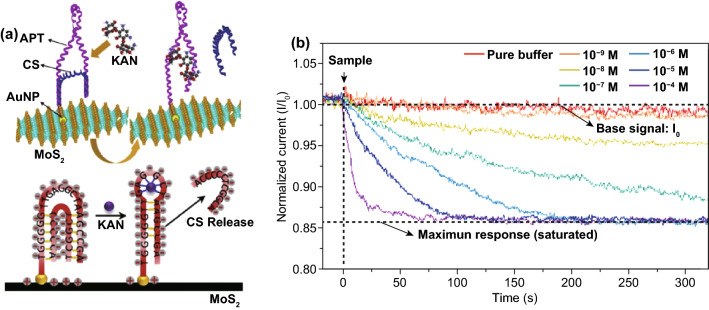
2D TMDC FET sensor for non-ionic molecule detection. **a** Sensing mechanism of kanamycin sensing on MoS_2_ FET platform. **b** Time-resolved responses for kanamycin at different concentrations. Reprinted with permission from Ref. [133], Copyright 2019, Elsevier

As FET sensor working in water is limited by the Debye screening effect, aptamers have been considered as a potential solution as sensing probe. A systematic study has achieved molecular recognition for antibiotics with MoS_2_-based aptasensor, demonstrating promising application of aptamer as probe on TMDC for the detection of neutral molecules [133]. As illustrated in Fig. 7a, the release of a complementary DNA strand from kanamycin aptamer was observed, offering a novel sensing mechanism. Different from the detection of charged molecules, this working principle that relied on charge release (the complementary DNA strand) from probe provided a methodology for electrically neutral molecule sensing and could overcome Debye screening limitation. Further investigation from dynamic sensing test demonstrated time-dependent sensitivity of the sensor (Fig. 7b), in which the ultratrace kanamycin detection capability is adjustable with merits of high stability and selectivity. Though there are limited studies on 2D TMDC-based FET sensors for non-ionic molecules in water, the fundamental knowledge and strategies provided by existing studies are significant for TMDC FET sensor design and fabrication, and can inspire future research on FET sensors for a variety of water contaminants.

#### Detection of Microorganisms

Microorganisms such as bacteria and virus can be threats for aquatic ecosystem and pathogens for human, and thus their existences in the water environment are highly concerned. Sensing strategies and devices for microorganism have been reported based on different principles and TMDC-based FET has recently become newly emerged sensing platform for microorganisms. The reports of TMDC FET sensors for the detection of various biomolecules including proteins [31, 32, 81, 134–143] and nucleic acids (DNA/RNA) [26–29, 144–149] demonstrated the superiorities of TMDC FET sensors in biomaterials sensing.

Moudgil et al. reported a highly sensitive and selective Gram-positive bacteria sensor based on a hybrid MoS_2_/TiO_2_ FETs [150], in which vancomycin with bio-affinity to *S. aureus* was functionalized onto MoS_2_ via a TiO_2_ layer, contributing to the efficient discriminating ability of the sensor between Gram-positive and Gram-negative bacteria. The detection of *Ebola*, a lethal virus, was achieved with FET sensors with liquid-phase exfoliated MoS_2_ [151], in which selective detection relied on the bio-recognition of a specific protein VP40 antigen on *Ebola*. Inspired by antibody–antigen interaction, the antibody of VP40 antigen was functionalized on the sensing surface as the receptor in this study. Generally, microorganism biosensing on TMDC FET mainly relies on a transducer to convert bio-recognition into detectable signals (i.e., TMDC channel) and a bio-receptor to achieve this bio-recognition behavior (i.e., detecting probe). The most critical but challenging work in microorganism detection on TMDC FET is the construction of TMDC channel with functional group or bio-receptor with specific affinity to target microbial cell or some characteristic components on the cell, as well as the surface functionalization methodology for the bio-receptor linking. As there is a range of threating microorganisms in water, further research in this area is needed to increase the sensing performance and applicability of TMDC FET sensors for microorganism detection.

To help get a clear understanding on recent advances of TMDC FET sensors for environmental analysis, comprehensive summaries of studies on TMDC FET sensors in gas sensing and water quality analysis are given in Tables 1 and 2, respectively. Various TMDCs with different layer structures and functionalization have been used in FET sensors that work in gaseous and aqueous media. Currently, most of the studies were carried out based on MoS_2_ because of the technical difficulty and high cost in the preparation of other TMDCs, as well as their chemical and electronic instability [152–154]. Strategies to lower the technical barrier and cost of material synthesis are needed for future development of 2D TMDC-based FET.

**Table 1:** Gas sensing with 2D TMDC-based FET sensors: device structure and sensing performance

Gas sensing		FET channel	LOD	Sensitivity (response)	Response/recovery time	References
Oxidizing gas	NO_2_	MoS_2_	20 ppb		–/60 min	[39]
		MoS_2_ (1L, CVD)	20 ppb	> 20%	10 min/–	[40]
		MoS_2_@CNT	1 ppm		366 s/1950s	[93]
		MoS_2_/SnO_2_	0.5 ppm	~ 0 6%	6.8 min/2.7 min	[12]
		MoS_2_/PtNP	2 ppb	3 (SNR)	> 30 min/> 30 min	[83]
		MoS_2_@WS_2_	50 ppm	26.12%	1.6 s/27.7 s	[107]
		MoS_2_/graphene	0.2 ppm	19%	6.5 s/6.5 s	[105]
		WS_2_ (4L)	25 ppm	8.7%		[15]
		WS_2x_Se_2-2x_		180% (10 ppm)		[103]
		WS_2_/AgNW	1 ppm	32%		[15]
		WS_2_/ZnS	5 ppm	32.5%	4 s/–	[106]
		WS_2_/WO_3_	100 ppm			[38]
		WS_2_/MOF	5 ppm	48.2%		[108]
		WSe_2_ (LPE)	50 ppb	5.06%	50 s /1050 s	[41]
		WSe_2_ (3L, ALD)	10 ppm		6.5 min/43 s	[98]
		MoTe_2_	12 ppb	1.15%	–/10 min	[42]
		α-MoTe_2_	70 ppb	13%	15 s/–	[43]
		MoSe_2_ (CVD)	10,300 ppm		–/30 min	[13]
		NbS_2_	241 ppb		3000 s/9000 s	[101]
	NO	MoS_2_ (1L)	0.3 ppm	50%	5 s/–	[88]
		MoS_2_ (2L)	0.8 ppm		> 2.5 min/–	[95]
	O_2_	MoS_2_ (3L, CVD)	100 ppm	4.84%	18 s/47 s	[155]
	SO_2_	MoS_2_/Ni	5 ppm	7.4%	50 s/56 s	[156]
Reducing gas	NH_3_	MoS_2_ (1L, CVD)	1 ppm	> 40%	5–9 min/–	[40]
		MoS_2_ (CVD)	300 ppb	4.2 (SNR)	15 s	[157]
		MoS_2_/ZnO	50 ppm	46.2%	10 s/11 s	[158]
		MoS_2_/TiO_2_ QDs	250 ppm	43.72%	–/ ~ 174 s	[159]
		WS_2_	5 ppm	–	120 s/150 s	[112]
		WS_2_	1.4 ppm	3.3 (SNR)	> 5 s/–	[111]
		WS_2_/WO_3_	1 ppm	1.16	15 min/28.9 min	[38]
		MoTe_2_	1 ppm	2.76%	–/10 min	[42]
		α-MoTe_2_	70 ppb	101%	1 s/2 s	[43]
		WSe_2_ (3L, ALD)	20 ppm		–/20 s	[98]
	H_2_	MoS_2_ (1L)	0.1%-90%		7 min/67 min	[160]
		MoS_2_/Si	1 ppm	30.4%	~ 400 s/ ~ 450 s	[36]
		WS_2_/PtNP	7.8 ppm	1.14	119 s/370 s	[37]
		WS_2/_WO_3_	1 ppm	1.15	5.9 min/27.2 min	[38]
VOCs	Acetone	MoS_2_	1 ppm		10 s/–	[18]
		MoS_2_/rGO	10 ppm		73 s/–	[161]
		WS_2_	5.6 ppm			[121]
		WS_2_ (2L)	0.5–10 ppm			[15]
		MoTe_2_	~ 0.2 ppm	3 (SNR)		[19]
	Triethylamine	MoS_2_ (1L)	1 ppm		15 s/30 s	[118]
		MoS_2_	1 ppm	~ 70%	~ 50 s/ ~ 100 s	[119]
		MoS_2_ (1T/2H contact)	80 ppb		~ 20 s/ ~ 50 s	[120]
	Ethanol	MoS_2_	10 ppm		10 s/-	[18]
		MoS_2_-MUA	100 ppm		10 s/-	[18]

*L* layer, *LPE* liquid-phase exfoliated, *ALD* atomic layer deposition, *QDs* quantum dots, *SNR* signal -to-noise ratio, *rGO* reduced graphene oxide, *MUA* mercaptoundecanoic acid

**Table 2:** Water quality analysis with 2D TMDC-based FET sensors: device structure and sensing performance

Water quality analysis		FET channel	Probe	LOD/sensitivity	Response time	References
Ions	H^+^	MoS_2_	HfO_2_	pH 3–9 (60.13 mV/dec)		[81]
		MoS_2_	Al_2_O_3_/HfO_2_	pH 0.01 (59.6 mV/dec)		[127]
		MoS_2_ (1L)	Ionic liquid-gate	–/(4.4 V/pH)		[162]
		MoS_2_/WS_2_/MoS_2_		pH 4–8 (59 mV/dec)		[128]
	Hg^2+^	MoS_2_		30 pM	~ 10 s	[20]
		MoS_2_	DNA	0.1 nM	1–2 s	[21]
	Cd^2+^	MoS_2_	Cd^2+^ ionophore	5 ng/mL	8 s	[129]
	AsO_3_^3−^	MoS_2_	CPPy	1 pM	< 1 s	[130]
	AsO_2_^−^	MoS_2_	AsO_2_^−^ ionophore	0.1 ppb	100–210 s	[22]
Non-ionics	H_2_O_2_	MoS_2_	rGO	1 pM	seconds	[24]
	Kanamycin	MoS_2_	DNA	1.06 nM	20 s	[133]
	Doxorubicin	MoS_2_	Cu^2+^-DNA		5 s	[132]
	Enkephalin	MoS_2_	MOR	~ 3 nM		[30]
Microorganism	Bacteria (*S. aureus*)	MoS_2_	Vancomycin	50 cfu/mL	22.19 s	[150]
	Virus (*Ebola*)	MoS_2_	VP40 antibody	fM-pM level	~ min	[151]

*L* layer, *MOR* µ-opioid receptor

## Conclusions and Outlook

2D TMDC is an emerging 2D nanomaterial group with a wide variety of individuals. Their physical and electronic properties including high surface-to-volume ratio, high carrier mobility, direct and tunable bandgap, and high transistor switching characteristic make them promising channel material in high-performance FET sensors. Considering the growing demand of environment analysis, in this article, we review recent advances of 2D TMDC FET sensors for gas and water contaminant detection with an emphasis on the working media and sensing mechanism. Working principles of TMDC FET sensors for the detection of various analytes in different media are discussed with a classification of target molecules based on the redox, where gaseous molecules are classified into oxidizing gas, reducing gas, and VOCs, while water contaminants are classified into anions, cations, and non-ionics. The sensing performance of TMDC FET sensors depends on various factors including 2D TMDC layer number, its intrinsic properties, contacting physics, and sensing probe. Different sensing mechanisms of TMDC FET sensors working in the air and in aqueous media are summarized based on the analysis of sensor structures and sensing capabilities. Surface functionalization is demonstrated as one of the key points in enhancing sensing selectivity and device stability especially in aqueous media.

In terms of future prospective of 2D TMDC-based FET sensors, there are plenty of room in sensor performance improvement for practical environment analysis. For gas sensors, though many types of TMDC channels with various physical/chemical modifications were reported and gas sensing capability were enhanced through a variety of research attempts, there is still a lack of a systematic understanding between the optimized performance and modification-induced geometrical, electronic, or chemical effects. Further studies are needed to offer more comprehensive understanding on physics and chemistry in material synthesis and sensor design for practical applications. For water contaminant sensing, the aqueous environment is one of the most challenging aspects at this early-stage research, though the reported achievements have inspired studies of more water contaminant species. The chemical and electrical impacts of surrounding water on TMDC channel require an in-depth study with a consideration for Debye screening, followed by more scientific attempts on the TMDC material construction and surface modification for sensitive and selective detection with a stable device structure.

Since most of the studies remain at the laboratory stage till now, though their potentials and superiorities in environmental analysis have been demonstrated, there are some critical challenges that need to be addressed to realize the sensor commercialization, from channel fabrication, device integration to detection capability. Specifically, the high cost and poor uniformity in the fabrication of 2D TMDC channels as well as the difficulties of their large-scale production are major obstacles in TMDC FET device fabrication. In addition, the signal analysis relies on professional semiconductor analyzer type of equipment, and thus on-site detection remains to be met with portable and affordable integrated device and detection system. Moreover, besides the sensing capability, the lack of detecting selectivity either in gaseous or aqueous surroundings is another barrier for immediate commercialization. All of the above are inevitable challenges to face and extensive studies are needed to achieve commercialization. Overall, TMDC-based FET is a promising sensor platform for highly effective environmental analysis, but wider and deeper studies are required for its practical applications. Further development of the sensors will rely on theoretical fundamentals for interactions between TMDC, sensing probe, analyte, and the working media.
